# Comparative Study of Systemic vs. Local Antibiotics with Subgingival Instrumentation in Stage III–IV Periodontitis: A Retrospective Analysis

**DOI:** 10.3390/antibiotics13050430

**Published:** 2024-05-09

**Authors:** Ioana Ilyes, Marius Boariu, Darian Rusu, Vincenzo Iorio-Siciliano, Octavia Vela, Simina Boia, Viorelia Radulescu, Petra Șurlin, Holger Jentsch, Alexandru Lodin, Stefan-Ioan Stratul

**Affiliations:** 1 Department of Periodontology, Faculty of Dental Medicine, Anton Sculean Research Center for Periodontal and Peri-Implant Diseases, “Victor Babes” University of Medicine and Pharmacy, 300041 Timisoara, Romania; ioana.veja@umft.ro (I.I.); rusu.darian@umft.ro (D.R.); vela.octavia@umft.ro (O.V.); simina.boia@umft.ro (S.B.); viorelia.radulescu@umft.ro (V.R.); stratul.stefan@umft.ro (S.-I.S.); 2 Department of Endodontics, Faculty of Dental Medicine, TADERP Research Center, “Victor Babes” University of Medicine and Pharmacy, 300041 Timisoara, Romania; 3 Department of Periodontology, University of Naples Federico II, 80131 Naples, Italy; enzois@libero.it; 4 Department of Periodontology, Faculty of Dental Medicine, University of Medicine and Pharmacy, 200349 Craiova, Romania; petra.surlin@umfcv.ro; 5 Medical Faculty, University of Leipzig, 04103 Leipzig, Germany; jenh@medizin.uni-leipzig.de; 6 Department Basis of Electronics, Faculty of Electronics, Telecommunications and Information Technology, Technical University of Cluj-Napoca, 400114 Cluj-Napoca, Romania; alexandru.lodin@bel.utcluj.ro

**Keywords:** antimicrobial resistance, systemic antibiotics, local antibiotics

## Abstract

To improve the clinical and microbiological outcomes of non-surgical mechanical periodontal therapy, the adjunctive use of antimicrobials has been utilized in treating moderate-to-severe periodontitis. In our study, the retrospective design included previously collected health-related patient data, obtained from the printed and digital charts of patients who received systemic or local antibiotic adjuncts to SI (subgingival instrumentation). A total of 34 patients (diagnosed with generalized Stage III/IV periodontitis) met the inclusion and exclusion criteria and were evaluated. The samples were tested for the following bacterial strains: *Aggregatibacter actinomycetemcomitans* (*A. actinomycetemcomitans*), *Porphyromonas gingivalis* (*P. gingivalis*), *Prevotella intermedia* (*P. intermedia*), *Tanererella forsythia* (*T. forsythia*), and *Treponema denticola* (*T. denticola*). The inter-group comparisons of the bacterial species did not show statistically significant differences between groups. The present study aimed to evaluate the clinical effects after SI and the adjunctive use of systemically administered (SA) AMX (amoxicillin) + MET (metronidazole) (administered for 7 days), with locally delivered (LDD) piperacillin + tazobactam in step 2 of periodontal therapy. Results: Overall, all parameters were improved in the groups, with a significant difference in inter-group comparison regarding the full-mouth bleeding score (FMBS) (*p* < 0.05) in favor of the SA group, and the *p*-value < 0.05 was considered to be statistically significant. Statistically significant PPD (probing pocket depth) reductions and CAL (clinical attachment level) gains were observed in both groups at the 3-month follow-up. In conclusion, within the limitations, the outcomes of this study suggest that SI, with adjunctive local or systemic antibiotic therapy, provided comparable clinical improvements. Systemic AMX + MET protocols were more efficacious with regard to the reduction in FMBS. Follow-up studies with larger patient numbers are needed to further investigate this effect.

## 1. Introduction

According to the Global Burden of Disease Study 2019, approximately 3.5 billion individuals worldwide are impacted by oral diseases, with periodontal diseases affecting roughly 50% of the global population [1]. Periodontal disease is a chronic multi-factorial inflammatory disease that affects the periodontium and is caused by the attachment of pathogenic bacteria to tooth surfaces. These bacteria form complex communities known as biofilms [2]. 

Standard periodontal treatment involves SI in removing biofilm and calculus from the affected root surfaces, which proved successful for the majority of patients [3,4]. Some limitations (inability to access deep pockets, surface irregularities, and furcation areas) required adjunctive antimicrobial agents in order to suppress or reduce the frequency of pathogenic bacteria [5]. To improve the clinical and microbiological outcomes of non-surgical mechanical periodontal therapy, the adjunctive use of antimicrobials has been indicated in treating moderate-to-severe periodontitis. Adjunctive antimicrobial therapy can be delivered either systemically or locally [6]. The literature describes the advantages and limitations of both forms of administration. Systemic administration offers the potential benefit of effectively targeting pathogens that have spread extensively throughout the oral cavity, including those in non-dental oral areas, such as the tongue’s upper surface and the tonsils’ crevices. Still, it requires a lot of patients to follow through with their treatment, can introduce unwanted systemic side effects that can make it harder for patients to do so, and may help bacteria become more resistant [7,8,9]. On the other hand, local administration is not influenced by patient adherence. The systemic method enables the direct application of drugs to the infected area at an unattainable concentration. However, their usage is restricted to specific clinically identified defects, and there is a possibility of reinfection from untreated regions or non-dental oral spaces [5].

In recent decades, antimicrobial resistance has become a pre-eminent concern among medical and public health professionals and a widespread global problem. The causative factors for this resistance remain uncontrolled, and national strategies must be developed to address the issue. The worldwide rise of antimicrobial resistance (AMR) poses a significant threat. As the World Health Organization (WHO) has mentioned, AMR contributes to almost 5 million human deaths from bacterial infections alone each year [10]. Because 10% of antibiotic prescriptions are from dentists [11,12,13], it is imperative to enhance the utilization of antibiotics to prevent the development of resistant bacteria linked to antibiotic treatments and the overuse of antibiotics [14]. The use of antibiotics should be strictly linked to evidence-based efficacy to reduce AMR, economic costs, and potential adverse effects [13]. Recently, the S3-level Clinical Practice Guideline (CPG) of the European Federation of Periodontology (EFP) for periodontitis Stages I–III offered an open recommendation for the adjunctive use of systemic antibiotics for specific patient categories (e.g., Stage III periodontitis in young adults) [14]. In this context, it is essential to optimize the antibiotic protocols when considering a minimal bactericidal concentration and a minimal duration to limit the side effects.

Amoxicillin, in combination with metronidazole, is considered to be an antibiotic regimen of first choice in severe periodontitis and is used widely [15,16,17]. The high efficiency of this combination for systemic use has turned it, in recent years, into a term of comparison with many other proposed antimicrobial or antimicrobial combination regimens (i.e., azithromycin, doxycycline, metronidazole, ornidazole, tetracycline) [18,19]. The combination of piperacillin–tazobactam is frequently used in the medical field; however, there are few studies in the dental field: one focused on odontogenic sinusitis [20], while a few recent studies have proven the adjuvant effect of the use of piperacillin–tazobactam in periodontitis [21,22,23]. Comparative evaluations of systemic antibiotics with local antibiotics are scarce—the last evaluation of the efficacy of systemic amoxicillin/metronidazole compared to the use of locally applied antibiotics with controlled release dates was from two decades ago [24]. Certain locally applied formulations, such as doxycycline (Ligosan^®^ Slow Release) and piperacillin + tazobactam (Gelcide^®^), are accessible in Europe and may be regarded as an adjunct in treatment for severe generalized periodontal disease. 

Over time, doubts have grown regarding the actual efficacy of these antibiotics when used locally. As a result, the majority of tested drugs have been removed from the market due to economic considerations or administrative barriers [6]. Many systematic reviews have documented additional advantages of using systemic antimicrobials in the treatment of periodontitis. The primary finding indicates that while there is enough evidence to suggest that systemic antimicrobials may be beneficial in treating periodontitis, there is now no ideal clinical strategy that can be suggested [15,18,19]. Despite the presence of strong and reliable data, there is insufficient support for well-defined clinical protocols, including specific products and dosages [6]; therefore, this retrospective study aimed to contribute to scientific evidence regarding the adjunctive use of local and systemic antimicrobials. Thus, the aim of the present study was to evaluate clinical effects after SI and the adjunctive use of systemically administered (SA) AMX + MET (administered for 7 days) or locally delivered (LDD) piperacillin + tazobactam during step 2 of periodontal therapy with respect to patients diagnosed with Stage III/IV periodontitis. This study also provides information on the superiority of these systemic antibiotics as adjuncts in non-surgical periodontal treatment compared to a novel, locally delivered, and slowly released combination of antibiotics to facilitate informed treatment decision-making. 

## 2. Results 

### 2.1. Characteristics of the Study Population 

Overall, 1363 charts of patients treated for severe periodontitis were screened. A total of 34 patients fulfilled the inclusion criteria; their demographic characteristics are summarized in Table 1. The study enrolled 13 women (F) and 21 men (M), ages ranging from 32 to 57, with a mean age of 45.62 ± 7.86 years. Among the enrolled patients, 20.58% (13.33% in group A and 26.31% in group B) were smokers. None of the enrolled patients reported any adverse events attributable to treatment. 

Table 2 shows the results of the Shapiro–Wilk test used to evaluate data distribution. The medians and interquartile ranges (IQRs) were calculated for full-mouth PPD and CAL (mm), as well as for FMPS and FMBS (%). There was no difference between the groups at baseline; no statistically significant inter-group differences could be detected with respect to gender, smoking, or the initial clinical periodontal parameters: PPD, CAL, FMPS, and FMBS.

### 2.2. Clinical Variables

**Figure 1:**
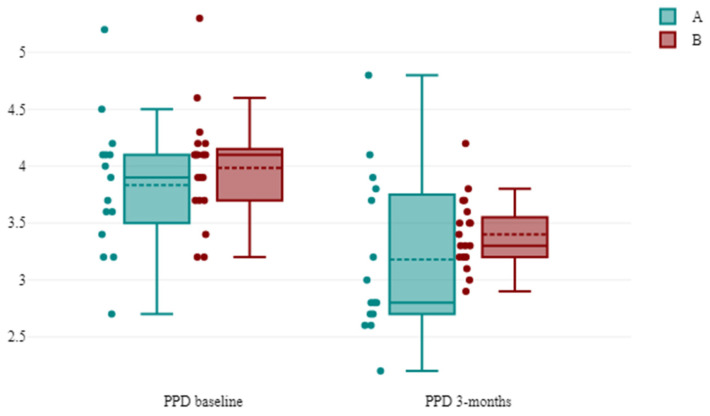
The evolution of the PPD between successive time points in the groups. Notes: A = group A systemic administration of AMX + MET; B = group B subgingival application of the piperacillin + tazobactam gel.

Table 3 shows the PPD evolution; the Mann–Whitney U test results suggest a small effect size (*r*-value = 0.159) in the difference between PPD at 3 months, with group A likely having lower values. The difference is not statistically significant at a 5% significance level, with a *p*-value = 0.104. Both treatments showed a statistically significant clinical decrease in PPD, *p* < 0.001 (Figure 1). 

**Figure 2:**
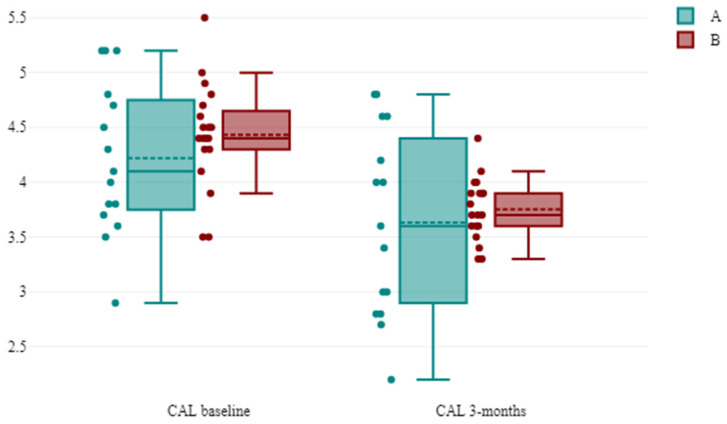
The evolution of the CAL between successive time points in the groups. Notes: A = group A systemic administration of AMX + MET; B = group B subgingival application of the piperacillin + tazobactam gel.

The mean values ± SD of CAL at baseline and 3 months are presented in Table 4. The mean baseline CAL was 4.22 ± 0.70 mm in group A (SA) and 4.43 ± 0.47 mm in group B (LA). At 3 months, the CAL value was 3.63 ± 0.86 for group A and 3.75 ± 0.28 in group B. The inter-group comparison revealed no statistically significant reduction (*p* > 0.05). In both groups, at 3 months, the values decreased statistically significantly, *p* < 0.05 (Figure 2).

**Figure 3:**
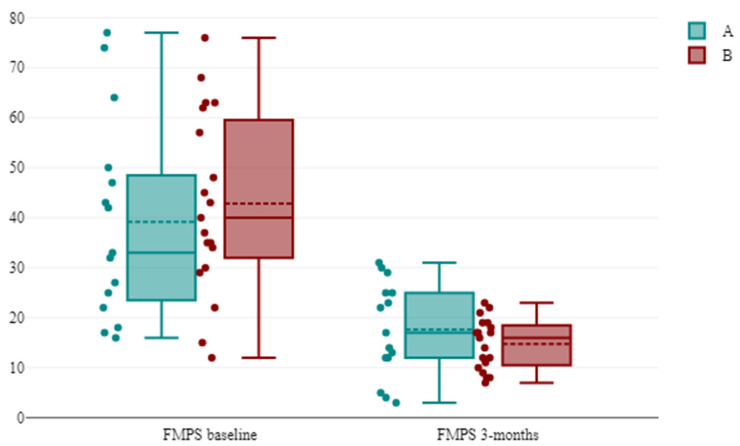
The evolution of the FMPS between successive time points in the groups. Notes: A = group A systemic administration of AMX + MET; B = group B subgingival application of the piperacillin + tazobactam gel.

**Figure 4:**
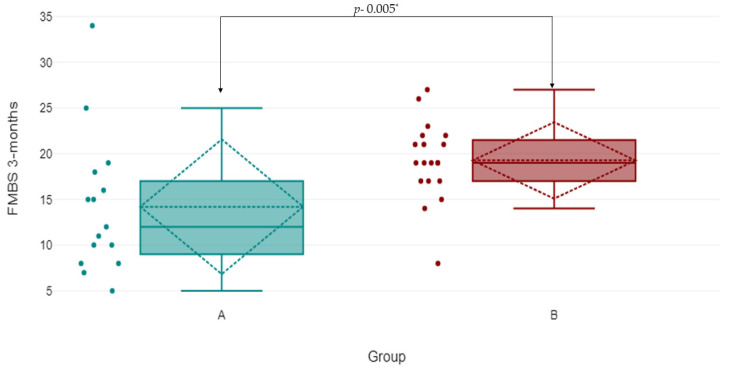
The evolution of the FMBS between successive time points in the groups. Notes: A = group A systemic administration of AMX + MET; B = group B subgingival application of the piperacillin + tazobactam gel. * *p*-value at 3-month examination, between groups.

The evolution of FMPS and FMBS is shown in Table 5 and Table 6; overall, both parameters were improved in all groups, with a significant difference regarding FMBS (*p* < 0.05). The results of the descriptive statistics showed that group A (SA) had lower values for the dependent variable (Mdn = 12) than group B (LA) (Mdn = 19). For the given data, a Mann–Whitney U test showed that the difference between groups with respect to the dependent variable FMBS was statistically significant and in favor of group A: *U* = 63.5 and *p* = 0.005. The effect size *r* was 0.4718, which is a medium effect. Thus, the null hypothesis was rejected (Figure 3 and Figure 4). 

Intra-group analysis between baseline and 3-month examination showed significant differences in all variables, which were statistically significant at a 5% significance level.

### 2.3. Microbiological Outcomes at Baseline 

Inter-group comparisons of the bacterial species *P. gingivalis*, *P. intermedia*, *T. forsythia*, *T. denticola*, and *A. actinomycetemcomitans* analyzed at baseline, if any were present in Stage III/IV periodontitis patients, did not exhibit statistically significant differences between groups (Table 7). A multiple linear regression analysis was performed to examine the influence of the frequency distributions of *A. actinomycetemcomitans*, *P. gingivalis*, *P. intermedia*, *T. forsythia*, and *T. denticola* on the variable PPD at 3 months. The model showed that the results explained 45.70% of the variance from the variable PPD at three months. An ANOVA was used to test whether this value differed significantly from zero. It was found that the effect was not significantly different from zero: *F* = 0.8946, *p* = 0.581, and *R*^2^ = 0.4571. A multiple linear regression analysis was performed to examine the influence of the microbiological results on the variable CAL at 3 months; the regression model showed that the results explained 40.98% of the variance from the variable CAL at 3 months. An ANOVA was used to test whether this value differed significantly from zero. Using the present sample, it was found that the effect was not significantly different from zero: *F* = 0.7378, *p* = 0.738, and *R*^2^ = 0.4098. No association could be determined between the clinical and microbiological characteristics in both groups. No significant differences were identified between individuals who smoked and those who did not smoke. 

## 3. Materials and Methods

The present retrospective study was conducted per the Declaration of Helsinki (World Medical Association, 1975), revised in 2013 (World Medical Association, 2013). The protocol was approved by the Committee for Research Ethics of the Victor Babes University of Medicine and Pharmacy Timisoara (approval No. 44/20.12.2023).

### 3.1. The Hypothesis 

No statistically significant differences will be observed with respect to the clinical parameters (e.g., BoP, PPD, FMBS, FMPS) between the two treatment modalities (i.e., the systemic use of AMX and MET administered for 7 days vs. locally delivered piperacillin + tazobactam protocol adjunctive to SI).

### 3.2. Sample Calculation

Based on the systematic review conducted by Herrera et al. in 2002, the use of additional systemic antibiotics alongside subgingival instrumentation (SI) may lead to an additional reduction of around 0.5 mm (with a range of 0.06–0.6 mm) in the average full-mouth pocket depth (PPD) compared to SI alone [19]. A difference of 0.6 mm between groups in the mean full-mouth PPD decrease after 3 months was considered clinically significant. With a common standard deviation of 0.5 mm for full-mouth PPD changes in both groups, the required sample size per group was 12 at 80% power to accurately detect a true difference. The sample size calculation was carried out using a two-tailed *t*-test of the difference between means with 80% power and a 5% level of significance.

### 3.3. Study Population and Data Extraction

The present study’s retrospective design included previously collected health-related patient data, respecting ethical research principles on humans and data confidentiality. The sample size was the data obtained during five years (2017–2022). Data were obtained from the printed and digital charts of patients who received conservative standard active periodontal therapy (APT) performed in a private practice in Timisoara, Romania, and the Department of Periodontology, Faculty of Dental Medicine “Victor Babes” University of Medicine and Pharmacy, Timisoara. The clinical diagnosis according to the New Classification for Periodontal and Peri-implant Diseases and Conditions (2018) [25] was retrospectively formulated for patients at baseline, using the 2017 World Workshop case definitions. A single experienced board-certified periodontist performed all diagnostic and treatment procedures. 

All patients whose records were selected for this study were examined by the same three examiners. The average intra-examiner calibration was 0.87, and the average inter-examiner calibration was 0.85, indicating a satisfactory agreement with the intra-class correlation coefficient (which was used to standardize data collecting and analyze research variables). 

**Figure 5:**
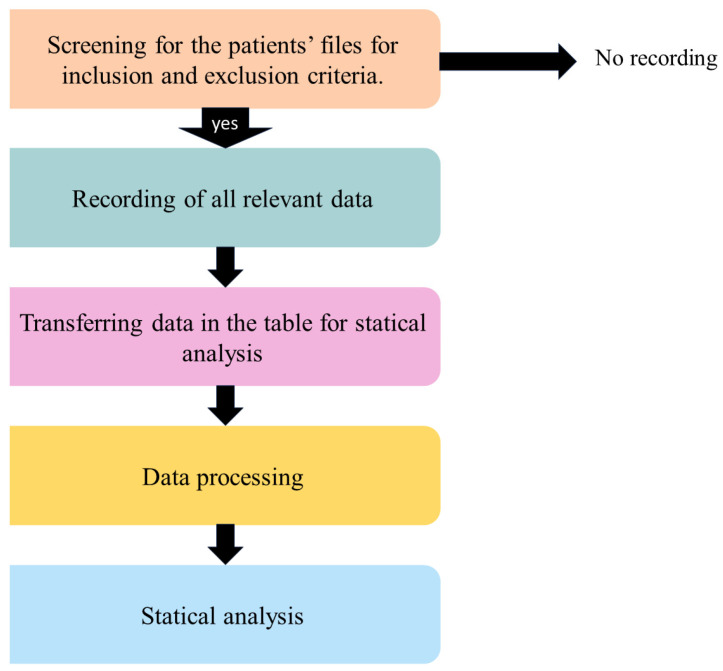
Flowchart of the data assessment and analyses.

A total of 34 systemically healthy adults were diagnosed with generalized Stage III/IV advanced and severe periodontitis. Figure 5 represents the flowchart of the data assessment and analyses. The data were recorded if a patient’s file met the inclusion criteria.

The inclusion criteria were as follows:

- Age ≥ 18 years old;
- Patients presented with untreated periodontitis (no supportive periodontal therapy and no periodontal treatment within five months);
- The clinical diagnosis of periodontitis Stage III–IV;
- Available microbiological analyses at baseline (before treatment) (*A. actinomycetemcomitans*, *P. gingivalis*, *T. forsythia*, *T. denticola*, and *P. intermedia*);
- At least ten natural teeth;
- Outcomes documented at baseline and post-treatment at 3 months.

The exclusion criteria were as follows:

- All vulnerable persons, defined as age less than 18 years;
- Pregnant women;
- Smoking >10 cigarettes/day;
- Patients with immune systemic disease;
- Individuals unable to consent were excluded;
- Systemic/local use of antibiotics within the previous 6 months;
- Additionally, patients who received periodontal surgery between the two evaluation time points were excluded.

Smoking habits were recorded in terms of current exposure (cigarettes/day), and patients were grouped as follows: light smokers (<10 cigarettes/day), moderate smokers (<20 cigarettes/day), and heavy smokers (≥20 cigarettes/day).

### 3.4. Clinical Evaluations

The probing pocket depth (PPD) was recorded at six points (three—mesial, central, and distal on both the buccal and the lingual/palatal side) measured from the mucosal margin to the bottom of the probable pocket to the nearest millimeter. The same type of manual periodontal probe (UNC15; Hu-Friedy, Chicago, IL, USA) was used, with light probing force applied. Bleeding on probing (BoP) was recorded as 0 (no bleeding) or 1 (bleeding) after probing for PPD [26] (presence/absence of bleeding within 30 s following probing), and plaque was assessed dichotomously. 

The clinical attachment level (CAL) was evaluated as the distance from the cementoenamel junction/restoration margin to the most apical point of the periodontal pocket. The full-mouth bleeding score (FMBS) represents the percentage of sites with bleeding on probing in all teeth (O’Leary, 1972) [27]. The full-mouth plaque score (FMPS) represents the percentage of sites covered with plaque in the entire dentition (Claffey 1990) [28]. Microbiological samples were collected from the deepest sites at baseline after removing the supragingival biofilm. 

### 3.5. Periodontal Treatment

The data were recorded after the patient’s file was checked and met the inclusion criteria. The recorded data included patient-related data (gender, age, presence of systemic diseases, and smoking status), clinical data (diagnosis; number of teeth; number of sites with PPD ≥ 5 mm; and BoP, CAL, FMBS, and FMPS); and the results of the microbiological analysis from baseline. 

The treatment protocol included the following: supra- and subgingival instrumentation (SI) under local anesthesia in all sites with PPD ≥ 4 mm using Gracey curettes (Hu Friedy, Chicago, IL, USA) and ultrasonics (EMS, Nyon, Switzerland), carried out by an experienced periodontist. The indicated oral health recommendations include brushing the teeth with either a manual or powered toothbrush for a minimum of 2 min, twice a day, and using interdental brushes for cleaning between the teeth. Instructions were tailored to the patient’s specific requirements for optimal plaque control. The participants were divided into two groups:

Group A (*n* = 15 patients): SI was followed by the systemic administration of AMX + MET, 500 mg, three times daily (TID) for 7 days;

Group B (*n* = 19 patients): SI was followed by a single subgingival application of the piperacillin + tazobactam gel in the same session.

**Figure 6:**
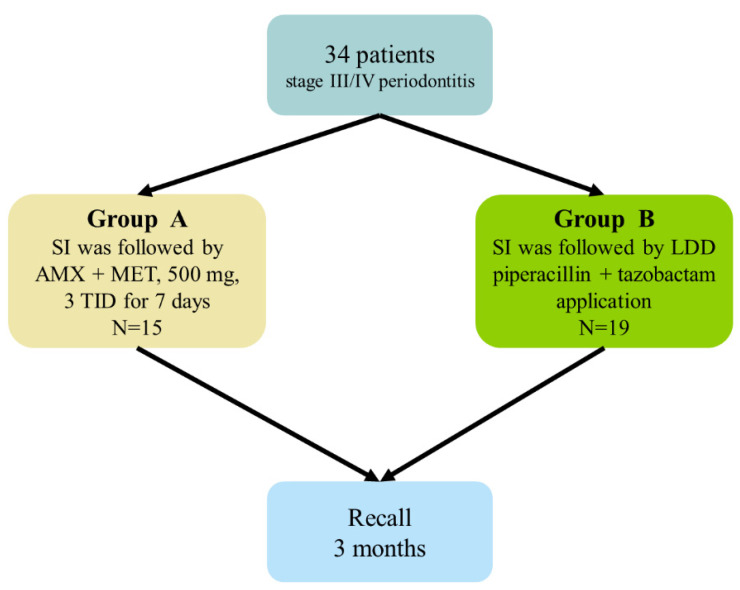
The study protocol.

Figure 6 summarizes the study protocol.

### 3.6. Microbiological Characterization at Baseline

During the initial investigation, microbiological samples were collected from sites at a minimum depth of 5 mm. Four sites were chosen, with one in each quadrant. These sites served as reference sites for the samples gathered at the beginning of the study. The subgingival plaque was sampled for microbiological evaluation as follows: The site was isolated with rolled wool, the overgrowth plaque was eliminated with a sterile compress, the gingival surface was dried, and plaque samples were obtained by inserting 2 sterile ISO #30 paper cones into the site, which were left in place for 30 s saturation [29]. The samples were tested for the following bacterial strains: *A. actinomycetemcomitans*, *P. gingivalis*, *P. intermedia*, *T. forsythia*, and *T. denticola*. Polymerase chain reaction (PCR) testing was conducted at the laboratories of the Department of Biochemistry of the “Victor Babeş”, University of Medicine and Pharmacy. 

After 15 min of vigorous mixing using a vortex at room temperature, the cones were taken out, and the liquid samples were purified by spinning them in a centrifuge for 5 min at a force of 3000 times the acceleration due to gravity; this was carried out at a temperature of 23 degrees Celsius. The samples were initially held at a temperature of 20 °C for one day and subsequently maintained at a temperature of 80 °C until a microbiological analysis was conducted within a maximum of 30 days. The molecular genetic examination of the samples revealed the presence of the main periodontopathogens. The existence of these microorganisms was evaluated using a commercially available test called micro-IDent^®^ (Hain Lifescience, Nehren, Germany). 

For the genetic identification of periodontopathogenic bacterial species, the QIAamp DNA Micro Kit (Qiagen GmbH, Hilden, Germany) was used for DNA extraction. The absolute yield and quality of the extracted DNA were evaluated using a NanoDrop ND-1000 spectrophotometer (Thermo Fisher Scientific Inc., Waltham, MA, USA). Bacterial levels were evaluated by utilizing a commercially available test kit system and semiquantitative methods (micro-IDent; Hain Lifescience GmbH, Carlsbad, CA, USA). Amplification was performed in a thermocycler (Thermo Fisher Scientific Inc.) and using HotStar Taq polymerase (Qiagen GmbH). The results were classified into the following categories: 0, nondetectable; (1) 10^4^ (10^3^ for Aa); (2) 10^4^–10^5^ (10^3^-10^4^ for A^a^); (3) 10^5^–10^6^ (10^4^–10^5^ for Aa); and (4) >10^7^ (10^6^ for Aa).

### 3.7. Data Analysis

The data from each patient were inserted into a spreadsheet and meticulously reviewed for any errors in data entry. The average PPD reduction at three months was considered the primary outcome, while changes in other periodontal clinical parameters (FMPS, FMBS, and average CAL) were secondary outcomes. Clinical periodontal parameters were evaluated at baseline and three months post-treatment, while microbiological variables were compared at baseline. The mean, median, and standard deviation (SD) were calculated for continuous variables. Frequencies and percentages were used to express data distributions for categorical variables. Clinical parameters were calculated as the full-mouth mean PPD, CAL, the full-mouth plaque score (FMPS), and the full-mouth bleeding score (FMBS). For statistical analyses, only the smoking status at baseline was used. The Shapiro–Wilk tests were used to evaluate data distribution. The normality of the distribution of the parametric data was assessed using the chi-square test. Differences between groups at baseline and 3-month visits and their changes were determined by the Mann–Whitney U tests for quantitative outcomes. For continuous data, intra-group comparisons were carried out using the Wilcoxon signed-rank test. Furthermore, clinical variables were compared with a repeated-measures ANOVA with post hoc Bonferroni’s corrections for inter-group and intra-group comparisons. The types of tests used are mentioned in each table’s footnotes. The results for all *p*-values of < 0.05 were considered statistically significant. For the statistical analysis, the DATAtab Team (2024) Statistics Calculator [30] was used.

## 4. Discussion

Antimicrobials have been indicated in the treatment of moderate-to-severe periodontitis. A common therapy approach, in this case, involves the use of local and/or systemic adjunct measures, such as chlorhexidine, hyaluronan, probiotics, and antibiotics, together with mechanical treatment [22,31,32]. The objective of the adjunctive antimicrobial’s use is to maximize the efficiency of infection management, limit the tissue damage due to the immune response, and optimize the healing process. However, the use of systemic antibiotic (SA) treatment is not commonly embraced due to concerns regarding the escalating issue of bacterial resistance [5] and the development of adverse effects, such as allergic/hypersensitivity events [33]. Therefore, it is recommended that the use of systemic antibiotics be reduced wherever possible, and the utilization of supplementary systemically administered antibiotics should be limited mainly to individuals who would obtain the greatest advantage from them [34,35,36]. Although adjunctive systemic antibiotics have been shown to be clinically effective in periodontal therapy, there are still some important concerns. The extensive use of antibiotics in medicine, often without a rational basis, and the excessive use of antibiotics in food production have led to a growing prevalence of bacterial resistance [37]. Patients have been prescribed local applications due to the reduced incidence of unwanted effects, decreased chance of developing bacterial resistance, and better compliance than compared to the use of systemic antimicrobials [6]. One of the main benefits of locally delivered antibiotics is the ability to administer lower quantities of topical drugs within the pocket, preventing the adverse effects associated with systemic antibacterial agents. This approach also enhances the exposure of certain microbes to elevated concentrations of the prescription, resulting in more effective therapeutic outcomes [38].

In general medicine, several areas have already implemented antimicrobial protocols for the responsible and appropriate use of antimicrobial agents [39]. In dental practice, the recent EFP S3-level clinical practice guideline (CPG) recommendations are for the rather restrictive use of adjunctive systemic antibiotics [14] and for patients with severe forms of periodontitis (≤36 years old or periodontitis with ≥35% of deeper sites (≥5 mm) and ≤56 years old) in order to provide extra clinically significant advantages [40]. For very advanced forms of periodontitis (Stage IV), no recommendations for adjunctive antimicrobial protocols exist so far. 

The present investigation aimed to determine if the systemic administration of AMX + MET for seven days, in addition to subgingival instrumentation (SI), is equivalent to treating Stage III/IV periodontitis with SI and locally administered piperacillin–tazobactam gel. It is widely supported in the literature on periodontal therapy that the systemic usage of AMX + MET, when used in conjunction with initial periodontal treatment in adult periodontitis patients, achieves significantly better clinical and microbiological results than initial periodontal treatment alone [18,41]. The current investigation results demonstrate that both treatments may lead to improvements in clinical parameters after a follow-up period of three months and provide relevant information on the management of periodontitis. Therefore, the use of the stated antimicrobials for local and systemic drug administration can effectively treat patients with severe generalized periodontal disease. This discovery, together with the objective of limiting antibiotic usage [42,43,44], indicates that the antimicrobial treatment of severe periodontitis with local adjuvants, such as the combination of piperacillin plus tazobactam, may be of clinical interest [45,46].

The available evidence in the field is limited; there are a small number of studies comparing systemically versus locally administrated antibiotics in periodontitis patients [24,37,47,48]. No such comparison using the mentioned antimicrobials has been published to date. The clinical treatment effects were evaluated at three months, as it is usually expected that the greatest advantages of SI with or without systemic or local adjunctive antimicrobials appear within the initial three months after treatment [49,50,51]. The primary outcome variable selected in our study was the mean difference in PPD reduction between the treatment groups at three months. The mean PPD reduction was 0.65 mm for the SA group and 0.60 mm for the local antibiotic (LA) group. These findings are in line with those who compared systemic versus local metronidazole administration in patients with periodontitis, where no difference regarding PPD reduction at six weeks was found between the SI + SA and SI + LA groups [47] with local tetracycline fibers and amoxicillin + acid clavulanic [24]. Another study with a longer follow-up period compared the LDD chlorhexidine chip and SA as adjuncts to SI in AgP and concluded that SA is more efficacious in clinical outcomes at six months [52]. In contrast, PPD was increased when administered in suppurating sites, which resulted in attachment loss [53]. The reduction in PPD and the attachment gain coincide with improvements reported in the literature, and the reduction is lower than that reported in the study of Duarte et al. [54] and higher than those of Mombelli et al. [52]. Interestingly, the local application of piperacillin–tazobactam from our study led to similar results with respect to tetracycline-loaded fibers used as an adjunct to SI, and it has shown clinical efficacy that is similar to SI combined with the systemic administration of amoxicillin/clavulanic acid in severe periodontitis in adult patients [24].

Our findings regarding changes in full-mouth CAL revealed that no statistically significant changes were observed within the treatment groups at three months. The improvements in clinical parameters in the SA group are consistent with the result of other studies, where PPD and CAL decreased at two and six months from baseline [55,56]. Moreover, in group LA, the decrease in clinical parameters was in agreement with previously reported data where the effects of LA (tetracycline fibers) evaluation suggested its adjunctive use to improve clinical response at two and six months after treatment [56]. Therefore, the additional administration of piperacillin–tazobactam may lead to a comparable result with 7 days AMX + MET after a follow-up period of 3 months in clinical parameters.

The systemic AMX + MET protocol was more efficacious with regard to the reduction in FMBS; in the SA group, the reduction was statistically significant. Moreover, these findings are in agreement with the above-mentioned studies [31,32,42]. Hence, the null hypothesis could not be rejected.

New studies are needed to address the current study’s limitations, which include a short evaluation period and the recruitment of a small percentage of severe periodontitis patients. A longitudinal assessment of these patients is essential to determine whether and to what extent SA and LA positively change clinical and microbial parameters over time. Another limitation of the present study is the lack of “negative control”. A “negative control” group treated merely with SI but without any antimicrobial medication may appear desirable for scientific purposes. The efficacy of adjuvant antimicrobial therapy in comparison to “SI alone” has been extensively documented for severe periodontitis and is well recognized in clinical practice [57,58]. Administering a less efficient treatment in a clinical study should be avoided, since it may provide ethical, acceptability, and feasibility concerns when compared to a more successful treatment (International Conference on Harmonization—ICH 2000). Therefore, our protocol has not included a “negative control” group (without any adjunctive antimicrobial therapy) [59]. The financial and time-consumption advantages of the one-time local antimicrobial treatment with the combination of piperacillin and tazobactam over the “standard” systemic administration of oral antibiotics have also not been evaluated in the present study.

## 5. Conclusions

In conclusion, within the limitations, the outcomes of this study suggest that SI, with the one-time adjunctive locally delivered piperacillin + tazobactam or the systemic AMX + MET antibiotic therapy, provided comparable clinical improvements. Statistically significant PPD reductions and CAL gains were observed in both groups at the 3-month follow-up. The systemic AMX + MET protocol was more efficacious with regard to the reduction in FMBS, and the null hypothesis was rejected. Follow-up studies with larger patient numbers are needed to further investigate this effect.

## Figures and Tables

**Table 1:** Demographic data in the groups at baseline.

Parameter	Group A	Group B	*p*-Value
*n*	15	19	
Age (years, mean ± SD) median	43.27 ± 9.18 42	47.47 ± 6.28 48	0.071 ^a^
Sex = female (*n*, %)	6 (40%)	7 (36.84%)	0.851 ^b^
Smoker (*n*, %)	2 (13.33%)	5 (26.31%)	0.353 ^b^

Abbreviations are as follows: *n*—number; SD—standard deviation; ^a^ Mann–Whitney U test; ^b^ chi-square test.

**Table 2:** Evaluation of data distributions at baseline.

Parameter	Group	N	Mean	SD	Min.	Max.	IQ	95 CI%	Mean ± SD	*p*-Value
Age	B	19	47.47	6.28	32	57	6	44.64; 50.29	47.47 ± 6.28	
	A	15	43.26	9.18	27	62	7	38.61; 47.91	43.26 ± 9.18	0.712 *
PPD	B	19	3.98	0.48	3.2	5.3	0.45	3.76; 4.20	3.98 ± 0.48	
	A	15	3.83	0.60	2.7	5.2	0.6	3.52; 4.13	3.83 ± 0.60	0.099 *
CAL	B	19	4.43	0.47	3.5	5.5	0.35	4.21; 4.64	4.43 ± 0.47	
	A	15	4.22	0.70	2.9	5.2	1	3.86; 4.57	4.22 ± 0.70	0.791 *
FMPS	B	19	42.84	18.11	12	76	27.5	34.69; 50.98	42.84 ± 18.11	
	A	15	39.13	20.12	16	77	25	28.94; 49.31	39.13 ± 20.12	0.147 *
FMBS	B	19	48.26	16.73	21	87	21	40.73; 55.78	48.26 ± 16.73	
	A	15	38.33	12.92	18	60	18.5	31.79; 44.87	38.33 ± 12.9	0.185 *

Abbreviations are as follows: IQ; interquartile range; SD: standard deviation; CI: confidence interval; N: number; PPD: probing pocket depth; CAL: clinical attachment level; FMBS: full-mouth bleeding score; FMPS: full-mouth plaque score; * Shapiro–Wilk test.

**Table 3:** Mean probing pocket depth (PPD) ± standard deviation (mm) at baseline and 3 months in the groups and *p*-values.

Variable	Group A		Group B		*p*-Value *
PPD	Mean ± SD	Median	Mean ± SD	Median	
BASELINE	3.83 ± 0.60	3.9	3.98 ± 0.48	4.1	0.372 *
3 MONTHS	3.18 ± 0.71	2.8	3.40 ± 0.31	3.3	0.104 *
DIFFERENCE TO BASELINE	0.65 ± 0.46	0.5	0.58 ± 0.28	0.6	0.945 *
*p*-value **	0.001 **		<0.001 **		

Abbreviations are SD: standard deviation; PPD: probing pocket depth; * Mann–Whitney U test; ** Wilcoxon test. Notes: *p*-values in bold indicate statistically significant differences in study groups.

**Table 4:** Mean clinical attachment level (CAL) ± standard deviation (mm) at baseline and 3 months in the groups and *p*-values.

Variable	Group A		Group B		*p*-Value *
CAL	Mean ± SD	Median	Mean ± SD	Median	
BASELINE	4.22 ± 0.70	4.1	4.43 ± 0.47	4.4	0.372 *
3 MONTHS	3.63 ± 0.86	3.6	3.75 ± 0.28	3.7	0.784 *
DIFFERENCE TO BASELINE	0.58 ± 0.41	0.5	0.67 ± 0.32	0.7	0.322 *
*p*-value **	0.001 **		<0.001 **		

Abbreviations are as follows: SD: standard deviation; CAL: clinical attachment level; * Mann–Whitney U test; ** Wilcoxon test. Notes: *p*-values in bold indicate statistically significant differences in study groups.

**Table 5:** Mean full-mouth plaque score (FMPS) ± standard deviation at baseline and 3 months in the groups and *p*-values.

Variable	Group A		Group B		*p*-Value *
FMPS	Mean ± SD	Median	Mean ± SD	Median	
BASELINE	39.13 ± 20.12	33	42.84 ± 18.11	40	0.537 *
3 MONTHS	17.66 ± 9.54	17	14.73 ± 5.05	16	0.302 *
DIFFERENCE TO BASELINE	21.46 ± 21.65	14	28.10 ± 16.08	26	0.147 *
*p*-value **	0.001 **		<0.001 **		

Abbreviations are as follows: SD: standard deviation; FMPS: full-mouth plaque score; * Mann–Whitney U test; ** Wilcoxon test. Notes: *p*-values in bold indicate statistically significant differences in study groups.

**Table 6:** Mean full-mouth bleeding score (FMBS) ± standard deviation at baseline and 3 months in the groups and *p*-values.

Variable	Group A		Group B		*p*-Value *
FMBS	Mean ± SD	Median	Mean ± SD	Median	
BASELINE	38.33 ± 12.92	39	48.26 ± 16.73	52	0.071 *
3 MONTHS	14.20 ± 7.62	12	19.26 ± 4.92	19	0.005 *
DIFFERENCE TO BASELINE	24.13 ± 10.17	29	29.00 ± 15.96	31	0.302 *
*p*-value **	0.001 **		<0.001 **		

Abbreviations are as follows: SD: standard deviation; FMBS: full-mouth bleeding score; * Mann–Whitney U test; ** Wilcoxon test. Notes: *p*-values in bold indicate statistically significant differences in study groups.

**Table 7:** Detection scores for the species *P. gingivalis*, *P. intermedia*, *T. forsythia*, *T. denticola*, and *A. actinomycetemcomitans* at baseline in the groups; data presented as frequencies (%).

Species	Timepoint	Detection Score	Group A	Group B	*p*-Value *
*A. actinomycetemcomitans*	baseline	0	11 (73.33%)	16 (84.21%)	0.758
		1	-	-	
		2	1 (6.66%)	-	
		3	3 (20.00%)	-	
		4	-	3 (15.78%)	
*P. gingivalis*	baseline	0	2 (13.33%)	1 (5.26%)	0.096
		1	12 (80.00%)	11 (57.89%)	
		2	1 (6.66%)	6 (31.57%)	
		3	-	1 (5.26%)	
		4	-	-	
*P. intermedia*	baseline	0	3 (20.00%)	6 (31.57%)	0.537
		1	10 (66.66%)	5 (26.31%)	
		2	2 (13.33%)	8 (42.10%)	
		3	-	-	
		4	-	-	
*T. forsythia*	baseline	0	-	-	0.071
		1	5 (33.33%)	1(5.26%)	
		2	5 (33.33%)	8 (42.10%)	
		3	5 (33.33%)	7 (36.84%)	
		4	-	8 (15.78%)	
*T. denticola*	baseline	0	1 (6.66%)	1 (5.26%)	0.077
		1	14 (93.33%)	11 (57.89%)	
		2	-	7 (36.84%)	
		3	-	-	
		4	-	-	

* Mann–Whitney U test.

## Data Availability

The data presented in this study are available upon request from the corresponding author.
